# Measuring Reasons for Unfinished Nursing Care as Perceived by Community Care Nurses: A Binational Development and Validation Study

**DOI:** 10.1111/phn.70140

**Published:** 2026-06-03

**Authors:** Stefania Chiappinotto, Einav Srulovici, Galit Palacios Klein, Nihal Khatib, Aysun Bayram, Luca Grassetti, Anat Drach‐Zahavy, Alvisa Palese

**Affiliations:** ^1^ Department of Medicine University of Udine Udine Italy; ^2^ The Cheryl Spencer Department of Nursing University of Haifa Haifa Israel; ^3^ Clalit Health Services Tel Aviv Israel; ^4^ Faculty of Health Sciences Department of Fundamentals Nursing Karadeniz Technical University Trabzon Türkiye; ^5^ Department of Economics and Statistics University of Udine Udine Italy

**Keywords:** community care, community health nurse, community nurse, reasons, unfinished nursing care

## Abstract

**Objective:**

To develop and validate the Unfinished Community Nursing Care Reasons Survey (UCNC_RS) transnationally and compare Unfinished Nursing Care (UNC) reasons across countries.

**Design:**

This development and validation study was conducted in Israel and Italy, following the COnsensus‐based Standards for the selection of health Measurement Instruments guidelines.

**Sample and measurement:**

Tool identification, adaptation, and development were followed by content and language validation; structural validation was then performed with 309 community nurses (156 in Israel and 153 in Italy) who agreed to participate. Exploratory Factor Analysis (EFA), Mokken analysis, and descriptive statistics were conducted.

**Results:**

The UCNC_RS consists of 23 items. Israeli EFA identified three factors, while the Italian EFA identified two, with items loading on more than one factor in both countries. Mokken analysis indicated a good model fit. Reasons for UNC differed between countries, both in their order of importance and in their averages, indicating different patterns of perceived elements affecting UNC occurrence.

**Conclusion:**

Overall, the UCNC_RS may be used in daily practice to survey and proactively address factors that may increase the occurrence of UNC. Reasons for UNC are organized hierarchically, suggesting that actions should target different system levels to reduce its occurrence. In Israel, priority policies are needed at the macro‐, exo‐, and nurse‐system levels, while in Italy, policies are needed at the macro‐, meso‐, and micro‐levels. Moreover, the reasons for UNC differ across countries, both in their order of importance and in their scores, with only some common elements, indicating different patterns of perceived reasons.

## 1 Background

Demographic and epidemiological changes worldwide have increased the need for new organizational models to ensure accessible care for vulnerable populations with chronic diseases. These models aim to prevent complications and control or slow disease progression by shifting care delivery from hospitals to patients’ homes and communities (Zeydani et al. 2023). In this context, community care refers to the provision of universally accessible, person‐centered, comprehensive health and social services delivered by a team of professionals accountable for meeting most health needs. These services are provided in sustained partnership with patients and caregivers, within the family and community setting, and play a central role in coordinating care and ensuring continuity (European Commission 2018).

Key professionals in this setting are Community Care Nurses (CCNs). They are responsible for proactively identifying individuals’ needs and implementing prevention programs at the individual, family, and community levels (European Commission 2018). CCNs provide a wide range of interventions both at home and in the community, focusing on health needs rather than solely on disease management (Dellafiore et al. 2022). As members of interdisciplinary teams, they collaborate with health and social care professionals, consistently placing individuals, families, and communities at the center of care (Dellafiore et al. 2022). The presence of CCNs in the community has been associated with improved quality of life, enhanced therapeutic adherence, better clinical outcomes, more efficient hospital use, and reduced avoidable admissions (Dellafiore et al. 2022). However, despite their key role, CCNs are not always able to provide the care required by patients, leading to the phenomenon of Unfinished Nursing Care (UNC).

Episodes of UNC within community nursing practice—defined as care required by individuals, families, or communities but left incomplete or delayed—have been documented (Jones et al. 2015). Initial investigations into UNC were conducted in Ireland (Phelan et al. 2018a; Phelan et al. 2018b; Rodríguez‐Vilas 2018) and the United Kingdom (Senek et al. 2020), where the most frequently reported unfinished activities included education and health promotion for vulnerable populations and older adults (Phelan et al. 2018a; Rodríguez‐Vilas 2018). UNC prevalence in these contexts has been reported to range from 32% to 70% of expected care (Phelan et al. 2018a; Senek et al. 2020).

However, despite its relevance, few studies have specifically focused on UNC among CCNs, with most attention given to home care nurses, whose scope of practice and roles differ (Di Nitto et al. 2024). In addition to quantifying UNC prevalence, it is essential to understand the underlying reasons for UNC, as perceived by CCNs, who in practice make both implicit and explicit decisions about which interventions are deprioritized or omitted. Existing literature has documented several contributing factors, including lack of administrative support, inadequate staffing levels, unexpected increases in patient numbers or acuity, and limited innovation or standardization in care approaches and technologies (Phelan et al. 2018a; Rodríguez‐Vilas 2018; Senek et al. 2020). Nurse‐specific factors such as age, education level, weekly working hours, and years of experience have also been associated with UNC (Phelan et al. 2018a; Rodríguez‐Vilas 2018). However, the tools validated to date (Palese et al. 2021) that include UNC reasons do not reflect the community context. For example, the Unfinished Nursing Care Survey (Bassi et al. 2020) includes reasons such as “Inadequate balance of nursing skills in the shift (e.g., excessive number of newly hired or non‐expert nurses compared to experts),” “Heavy admission/discharge activity during the shift (e.g., from/by hospital),” “Other departments did not provide the expected service (e.g., delay in diagnostic processes),” and “Nurse aides missed or delayed reporting the tasks left undone,” which clearly describe hospital care. Using such tools may provide inaccurate and irrelevant information and may delay the appropriate identification of strategies to mitigate the phenomenon.

Second, all tools measuring UNC at the community level have been validated in a single country (Phelan et al. 2018a; Phelan et al. 2018b; Rodríguez‐Vilas 2018; Senek et al. 2020), limiting their usability in cross‐national studies aimed at benchmarking incidence, reasons, and, above all, the effectiveness of preventive or mitigating strategies. Accurately measuring the reasons for UNC is essential for enabling nurse managers to implement effective preventive or mitigation strategies. Moreover, at the meso‐ and macro‐levels, evidence on the causes of UNC can inform broader policy decisions and planning efforts (Chiappinotto et al. 2022; Chiappinotto et al. 2023; Jones et al. 2019).

Third, with the rapid development of the CCN role worldwide (Lokhande 2023), developing a transnational tool may accelerate the production of evidence on reasons: as documented recently, the tools validated in the field of UNC (Palese et al. 2021) have accumulated evidence from one nation to another, and no studies have been designed based on a simultaneous process of tool development and validation. Establishing such a transnational tool would inform effective interventions at local, national, and international levels. Fourth, tool development should carefully consider the evidence produced in a given field, building new instruments upon those already well established. Therefore, instead of creating new tools, considering and further developing those already validated may increase the speed of evidence production (Palese 2026).

Taking all these elements into account, the intent of this study was to advance knowledge about the reasons for UNC as perceived by CCNs. The primary aim was to develop and validate a transnational tool for measuring UNC reasons; the secondary aim was to compare the reasons for UNC across countries.

## 2 Methods

### 2.1 Design and Setting

A development and validation study were conducted in accordance with the COnsensus‐based Standards for the selection of health Measurement INstruments guidelines (Mokkink et al. 2018) (Table S1). The study took place in Israel and Italy, where the roles of CCNs have been defined by specific policies (Table S2). These countries were chosen for their contrasting yet complementary healthcare systems and cultural contexts: Israel has a centralized, publicly funded healthcare system, whereas Italy's regionalized structure results in significant variation in community nursing practices (Fantozzi et al., 2025). Both countries face similar demographic challenges, such as ageing populations and increasing demand for community‐based care, but differ in cultural expectations and professional norms, which may affect the prioritization of care tasks. Furthermore, differences in policy frameworks and nurse education pathways offer valuable insight into systemic factors contributing to UNC. These differences and shared challenges make Israel and Italy well suited to exploring the cross‐national relevance of UNC reasons and informing strategies applicable across diverse healthcare systems.

### 2.2 Study Phases

Three phases were followed: (1) tool identification; (2) tool adaptation and development, content and language validation; and (3) structural validation.

1. Identification of the tool measuring UNC reasons

A rapid review of PubMed and CINAHL was conducted using the following keywords: “missed nursing care”, “task left undone”, “unfinished nursing care”, “implicit rationing of nursing care”, “tool”, “instrument”, “assessment”, and “community setting”. All literature on UNC measurement and community nursing care (Andersson et al. 2022; Phelan et al. 2018a; Phelan et al. 2018b; Senek et al. 2020; Sworn and Booth 2020) was examined. The MISSCARE Survey (Kalisch and Williams 2009) emerged, comprising 44 items listing all possible reasons, as used by Phelan et al. 2018a (Cronbach's alpha [*α*] > 0.70 for sections B, C, and D—the last measuring factors affecting missed care). However, although influential, the tool was not considered fully suitable by team members in the Israeli and Italian community care settings, even in its adapted form (Rodríguez‐Vilas 2018). Additionally, the tool used by Senek and colleagues (2020) was identified as employing a single‐measure question on a 5‐point scale to assess UNC, without evaluating its causes, and was therefore not suitable for our purpose. Researchers then considered the most recent validated tool, the Unfinished Nursing Care Survey (UNCS, Bassi et al. 2020), developed for hospital contexts, which demonstrates good psychometric properties (Part A: Rho = 0.96; Part B: *α* = 0.806) and represents the latest generation of tools in the field (Palese et al. 2021). The UNCS includes 18 items assessing all possible reasons for UNC, rated on a 4‐point Likert scale from 1 “not significant” to 4 “very significant reason”. The tool was considered the most suitable point of reference due to its (a) capacity to reflect UNC reasons and (b) adaptability and development in community care settings in both countries. The other tools (e.g., Andersson et al. 2022; Phelan et al. 2018a; Phelan et al. 2018b; Senek et al. 2020; Sworn and Booth 2020) were regarded as possible sources of insights and for checking completeness. The decisions made regarding tool identification were agreed upon by all members of the international team (see authors).

2. Tool adaptation, development, content and language validation

The research team adapted the UNCS (Bassi et al. 2020) for the community care setting in English. The adaptation process involved several monthly meetings from May to July 2022, during which each reason in the UNCS was discussed, compared with the specific community nursing literature (Andersson et al. 2022; Phelan et al. 2018a; Phelan et al. 2018b; Senek et al. 2020; Sworn and Booth 2020) and general literature (Chiappinotto et al. 2022; Chiappinotto et al. 2023), reformulated when necessary, further developed, and assessed for coherence with the community care systems in the two countries. The process included five nurses and an organisational psychologist with diverse backgrounds in research and community care. Consultations outside the research team (e.g., with expert nurse managers in community care and policy documents) also supported the achievement of a comprehensive representation of the core reasons for UNC in both countries at the community care level. Consensus on the items to be included in the tool was reached through discussion and agreement by the entire research group. Every disagreement was discussed in meetings until the group reached a final decision. The process, thoroughly documented at each step and co‐led by members from both countries, resulted in a tool comprising 23 items: seven were retained from the original 18 in the UNCS, four were slightly redefined, seven were deleted, and 12 were added (Table S3). The response options were on a 4‐point Likert scale from “Not significant reason” to “Very significant reason”, with an additional option for reasons not related to the specific context (“Not applicable”).

The UNCS Nurse Form was also adapted to include relevant sociodemographic data (e.g., gender, age, education, years of experience as a nurse and in the community) and information about the specific context of community care (e.g., shift profile, working overtime, number of days or shifts missed due to illness or injury in the last three months, shifts changed due to service needs, perceived adequacy of nursing resources and nursing aides). The adaptation process was the same as for the reasons section. Regarding nursing aides, the research group decided to remove this item from the tool because they were not present in the community care settings considered. However, the item was retained in the Nurse Form to assess whether nurses perceived a need for nursing aides, thereby suggesting a potential area of improvement in these settings.

The resulting Nurse Form and Unfinished Community Nursing Care Reasons Survey (UCNC_RS) tool were then translated into Italian and Hebrew. Language equivalence was ensured by following the four steps established by the World Health Organization translation guidelines (Younan et al. 2019): (a) translation—two researchers in each country independently translated the tool and then reached agreement; (b) expert committee assessment—experts (see authors) reviewed the original English and the initial translated versions in each country's language to assess content validity; (c) back translation—certified experts retranslated both versions into English to identify inconsistencies; and (d) pilot testing, which involved 30 community nurses in Italy and 33 in Israel, to assess the tool's feasibility, comprehensibility, clarity, and consistency with daily practice. The tool's instructions were also evaluated. Suggestions were made to change some terms (e.g., from “Other departments did not provide the service expected” to “Other multidisciplinary teams did not provide the service expected”). The tool was then considered ready for the validation phase.

3. Structural validity

Structural validity was assessed in 2024 (from July to September). As the UCNC_RS comprised 23 items, approximately 230 CCNs were expected (DiIorio 2006). However, to increase the study's power, the target population was set at 300 CCNs (Israel: 150; Italy: 150). Eligible participants were nurses (i) working in community settings and (ii) willing to participate. Nurses working in nursing homes, home care nurses, pediatric and/or palliative or community care nurses, or those not involved in patient care (e.g., nurse managers) were excluded.

Community care services accessible to researchers in both Israel and Italy were approached. Each country included all community services linked to the trust (Clalit Health Services) in Israel and (Azienda Sanitaria Universitaria Friuli Centrale) in Italy. Formal authorisation was requested from these services, after which nurse managers were contacted to explain the study procedures and aims. A link containing the full description of the study aims and procedures, with the consent form displayed on the first page, was distributed to eligible CCNs. After describing the concept of UNC, the main question was: “Can you indicate among the following list the reasons which, in your opinion, determine the UNC in your daily practice?” Online administration via EUSurvey and Nemala Plus required completion of all items of the UCNC_RS, while completion of the Nurse Form was optional. In total, 12 nurses did not fully complete the Nurse Form. Two reminders were sent, one at the beginning of data collection and one after a month, and the survey closed after two months. As completion of all items of the UCNC_RS tool was mandatory, no missing data were found.

Studies on UNC in the community care setting were analyzed to determine the best approach for structural validity. A scoping review of UNC in community settings, including 18 studies mainly conducted in residential care and nursing homes, identified organisational, staffing, and material insufficiencies, as well as issues related to working climate and staff. However, these studies did not provide a conceptual framework (Andersson et al. 2022). Primary studies documenting factors triggering UNC in community care, such as nurse age and poor administrative support, were also examined; as with the scoping review, no reference framework of reasons emerged (Phelan et al. 2018a; Senek et al. 2020; Sworn and Booth 2020). Variations in reasons across countries and studies have been reported, indicating the importance of contextual factors (Phelan et al. 2018a).

Given the absence of a conceptual framework for the reasons for UNC in community nursing care, an exploratory factor analysis (EFA) was conducted to align the unobserved factor structure with the data set. Initially, the EFA was explored in both data sets (Table S4). Given the findings, with cross‐loadings of approximately 47% (11 items out of 23), the process was then conducted at the country level, resulting in two separate EFAs. Factors were identified using the scree plot, and the complexity index was analysed to detect scores greater than 1, indicating that items load on more than one factor. The EFA at the country level was further justified for the following reasons: first, although the research team agreed on the items' content validity, UNC reasons may be context‐sensitive. Second, this comparison enabled exploration of potential differences in the underlying reasons for UNC at the construct level.

To determine the number of factors, Cattell's scree test (Cattell 1966) was used, as summarized in the scree plot. The plot displays the eigenvalues of a correlation matrix and the eigenvalues from a factor analysis; the representation is based on the Very Simple Structure criterion (Revelle and Rocklin 1979). This solution represents a compromise between improved fit measures and complexity indexes. The limited sample size makes other comparisons less reliable. Cronbach's alpha was calculated for the individual factors, and McDonald's omega (McDonald 1999) was calculated for the full tool.

Based on the findings, a Mokken analysis (Mokken library; Van der Ark 2007, 2012) was conducted to assess differences in the order of reasons given for UNC (Sijtsma and van der Ark 2017). The goodness of fit of the model was assessed by computing Loevinger's H coefficient (scalability); the scale was considered weak if 0.30 ≤ H < 0.40, moderate if 0.40 ≤ H < 0.50, and strong if H > 0.50. Invariant item ordering was assessed using the HT coefficient; similarly, invariant item ordering was considered weak if 0.30 ≤ HT < 0.40, moderate if 0.40 ≤ HT < 0.50, and strong if HT > 0.50.

Descriptive statistical methods, including mean, standard deviation, 95% confidence interval, frequencies, and percentages, were also used. Conditional averages with 95% confidence intervals and a comparison of medians using the Wilcoxon test were assessed between countries.

All analyses were conducted using R Statistical Software (R Core Team 2025) and the psych library (Revelle 2025) to perform EFA.

### 2.3 Ethical Considerations

The study was approved by the Ethical Board of University of Haifa, Israel and by the Institutional Review Board of the Medical Department of Udine University, Italy. Written permission from the original author of the UNCS was obtained. Participation was voluntary, and CCNs were informed of the aims and procedures of the study at the country level by a member of the research team. Data were anonymized to ensure confidentiality of participants and the community settings where they were working at the time of the study.

## 3 Results

### 3.1 Participants

A total of 309 CCNs participated, with 156 in Israel and 153 in Italy (Table 1), with response rates exceed 85% in each country. Participants were predominantly female (overall: 76.70%; Israel: 70.59%; Italy: 82.69%; *p* = 0.054), with a mean age of 45.71 years (standard deviation [SD]: 9.58) (Israel: 46.49, SD 9.57; Italy: 45.03, SD 9.56; *p* = 0.181). Overall, 24.60% of nurses were educated at the bachelor's level. In Israel, nurses were mainly educated at the master's or postgraduate level (47.71%), whereas in Italy, the Bachelor of Nursing Degree (35.26%) followed by the Nursing Diploma (34.61%) was most common (*p* ≤ 0.01). Nearly all participants reported more than one year of experience (Israel: 94.77%; Italy: 98.72%) and experience in the community setting (Israel: 91.50%; Italy: 94.87%; *p* = 0.665).

**TABLE 1:** Characteristics of the participants’ community care nurses.

Variable	Israel (*n* = 153)	Italy (*n* = 156)	Overall (*n* = 309)	*p*‐value
Gender, n (%)				
Female	108 (70.59)	129 (82.69)	237 (76.70)	0.054
Male	39 (25.49)	26 (16.67)	65 (21.04)	
Missing	6 (3.92)	1 (0.64)	7 (2.26)	
Age, mean (SD)	46.49 (9.57)	45.03 (9.56)	45.71 (9.58)	0.181^*^
Education, n (%)				
Nursing diploma	13 (8.50)	54 (34.61)	67 (21.68)	<0.001
Bachelor degree	21 (13.73)	55 (35.26)	76 (24.60)	
Bachelor degree + postgraduate course	24 (15.69)	38 (24.36)	62 (20.06)	
Master degree	9 (5.88)	1 (0.64)	10 (3.24)	
Master degree + postgraduate course	73 (47.71)	2 (1.28)	75 (24.27)	
Other	9 (5.88)	6 (3.85)	15 (4.85)	
Missing	4 (2.61)	0 (0.00)	4 (1.30)	
Experience as a nurse, n (%)				
<1 year	1 (0.65)	2 (1.28)	3 (0.97)	‐^**^
>1 year	145 (94.77)	154 (98.72)	299 (96.76)	
Missing	7 (4.58)	0 (0.00)	7 (2.27)	
Experience in the community care setting, n (%)				
<1 year	5 (3.27)	8 (5.13)	13 (4.21)	0.665
>1 year	140 (91.50)	148 (94.87)	288 (93.20)	
Missing	8 (5.23)	0 (0.00)	8 (2.59)	
Shift type, n (%)				
Day shift	111 (72.55)	117 (75.00)	228 (73.79)	<0.001
Morning only	39 (25.49)	17 (10.90)	56 (18.12)	
Afternoon only	0 (0.00)	0 (0.00)	0 (0.00)	
Other	2 (1.31)	22 (14.10)	24 (7.77)	
Missing	1 (0.65)	0 (0.00)	1 (0.32)	
Overtime in last three months, n (%)				
Yes	71 (46.41)	122 (78.20)	193 (62.46)	<0.001
No	76 (49.67)	34 (21.80)	110 (35.60)	
Missing	6 (3.92)	0 (0.00)	6 (1.94)	
Shifts missed due to illness/injury in last 3 months, n (%)				
None	75 (49.02)	102 (65.39)	177 (57.28)	0.002
1 day or shift	31 (20.26)	10 (6.41)	41 (13.27)	
2–3 days or shifts	27 (17.65)	21 (13.46)	48 (15.53)	
4n6 days or shifts	9 (5.88)	11 (7.05)	20 (6.47)	
More than 6 days or shifts	7 (4.58)	12 (7.69)	19 (6.15)	
Missing	4 (2.61)	0 (0.00)	4 (1.30)	
Shift changes due to service needs in the last month, n (%)				
No changes in shifts	47 (30.72)	75 (48.08)	122 (39.48)	0.001
Less than 3 shifts	66 (43.14)	70 (44.87)	136 (44.01)	
From 3 to 5 shifts	26 (16.99)	10 (6.41)	36 (11.65)	
More than 5 shifts	8 (5.23)	1 (0.64)	9 (2.91)	
Missing	6 (3.92)	0 (0.00)	6 (1.95)	
Adequacy of nursing resources, n (%)				
0% of the time	7 (4.57)	2 (1.28)	9 (2.92)	<0.001
25% of the time	16 (10.46)	10 (6.41)	26 (8.41)	
50% of the time	36 (23.53)	68 (43.59)	104 (33.66)	
75% of the time	66 (43.14)	67 (42.94)	133 (43.04)	
100% of the time	25 (16.34)	9 (5.78)	34 (11.00)	
Missing	3 (1.96)	0 (0.00)	3 (0.97)	
Adequacy of nurse aides’ resources, n (%)				
0% of the time	67 (43.79)	18 (11.54)	85 (27.51)	<0.001
25% of the time	30 (19.61)	28 (17.95)	58 (18.77)	
50% of the time	17 (11.11)	49 (31.41)	66 (21.36)	
75% of the time	16 (10.46)	61 (39.10)	77 (24.92)	
100% of the time	11 (7.19)	0 (0.00)	11 (3.56)	
Missing	12 (7.84)	0 (0.00)	12 (3.88)	

Abbreviations: *n*, number; SD, Standard Deviation; *p*‐values for the Chi‐Squared test for the association.

^*^ *p*‐value for the t‐test for the comparison of the mean

^**^ *p*‐value not available.

At the time of the study, most nurses worked mainly in daily shifts (overall: 73.79%), with the proportion slightly lower in Israel than in Italy (Israel: 72.55%; Italy: 75.00%; *p* ≤ 0.001). The majority reported working overtime in the last three months (62.46%), with a lower percentage in Israel (46.41%) compared to Italy (78.20%) (*p* < 0.001). More than half reported missing one or more shifts due to illness or injury (42.72%), with a higher percentage in Israel (50.98%) compared to Italy (34.61%) (*p* = 0.002). Most nurses reported shift changes due to service needs (60.52%), more frequently in Israel (69.28%) than in Italy (51.92%) (*p* = 0.001).

The perceived adequacy of nursing resources was reported as sufficient 75% to 100% of the time by 54.04% of participants, with a higher proportion among Israeli nurses (59.48%) then Italian nurses (48.72%) (*p* < 0.001). In contrast, the adequacy of nursing aides was perceived as poor, with 67.64% of participants reporting adequacy only 0% to 50% of the time (Israel: 74.51%; Italy: 60.90%; *p* ≤ 0.001).

### 3.2 Unfinished Community Nursing Care Reasons Survey

Overall, the UCNC_RS tool, consisting of 23 items, was validated for content and language. The EFA conducted on Israeli data identified three factors, while the Italian data revealed two factors (Table 2; Figures S1 and S2). In the Israeli EFA, Factor 1, “Care Coordination and Resource Availability Challenges,” covers issues related to the organization, communication, and availability of resources in nursing care (explained variance = 29.0%). Factor 2, “Challenges in Nursing Workflow and Care Prioritization,” refers to everyday operational difficulties faced by nursing teams that hinder the smooth delivery of care. Key issues include staffing shortages, uneven workloads, sudden increases in patient needs, task disruptions, and care models that do not adapt well to changing conditions (explained variance = 19.9%). Factor 3, “Nursing Skill Mix and Competence Alignment,” addresses the adequacy and distribution of nursing skills within a team, focusing on the balance between advanced and basic competencies (explained variance = 16.7%). Overall, the model explains 65.6% of the total variance, with an internal consistency of 0.925.

**TABLE 2:** Explorative factor analysis of Israeli and Italian data.

	Israel				Italy		
Items	Factor 1	Factor 2	Factor 3	Complexity	Factor 1	Factor 2	Complexity
1. Inadequate number of nurses	0.239	0.659	−0.107	1.374	0.459	0.340	1.843
2. Inadequate number of administrative personnel	0.360	0.490	−0.007	1.841	0.364	0.185	1.487
3. Inadequate skill‐mix (e.g., excessive number of nurses without advanced training compared to nurses with advanced training)	−0.042	−0.143	0.745	1.066	0.604	0.251	1.335
4. Inadequate nursing care model (e.g., functional tasks‐oriented model of care)	0.443	0.593	0.091	1.793	0.659	0.232	1.244
5. Unbalanced workloads assigned across nurses during the shift	0.293	0.767	0.006	1.272	0.561	0.343	1.656
6. Unbalanced nursing competences in the shift (e.g., excessive number of newly hired or non‐expert nurses compared to experts)	−0.112	0.089	0.797	1.073	0.643	0.242	1.278
7. Unexpected rise in patient acuity/complexity	0.230	0.607	−0.137	1.321	0.439	0.400	1.983
8. Increased workload during the shift (e.g., increased numbers of patients at need)	0.273	0.746	−0.201	1.425	0.465	0.441	1.994
9. Inaccurate initial priority setting	0.596	0.545	−0.003	1.998	0.692	0.311	1.388
10. Inadequate priority re‐assessment during the shift	0.561	0.584	0.111	2.071	0.751	0.292	1.295
11. Inadequate skills/competences of nurses	0.092	−0.035	0.905	1.025			1.539
12. Inadequate confidence in performing skills by nurses	0.068	−0.174	0.850	1.097	0.670	0.362	1.827
13. Underuse or under recognition of the nursing competences	0.124	−0.122	0.844	1.088	0.634	0.461	1.606
14. Repeated interruptions of care activities (e.g., unplanned or unexpected activities)	0.434	0.602	−0.164	1.900	0.617	0.358	1.591
15. Medications prescribed not available	0.771	0.359	0.039	1.486	0.585	0.334	1.391
16. Equipment not available/not functioning properly when needed	0.712	0.440	0.053	1.730	0.277	0.614	1.628
17. Other multidisciplinary team did not provide the service expected (e.g., delays in obtaining the home advice for palliative care, mental health care)	0.654	0.523	−0.041	1.953	0.376	0.632	1.297
18. Caregiver unavailable (e.g., to inform him/her about the care to be continued at home)	0.708	0.394	−0.105	1.654	0.302	0.776	1.139
19. Incomplete or interrupted communication between the community nursing staff and the hospital nursing staff	0.779	0.329	−0.039	1.388	0.212	0.802	1.723
20. Incomplete or interrupted communication among the community nursing staff	0.867	0.192	0.086	1.130	0.395	0.604	1.843
21. Incomplete or interrupted communication between nursing and medical staff	0.896	0.237	−0.000	1.162	0.495	0.668	1.514
22. Lack of support/collaboration among the community team members	0.835	0.318	−0.012	1.324	0.339	0.644	1.995
23. Excess time spent travelling to reach homes or the community centers	0.559	0.217	0.315	1.907	0.529	0.556	1.645
Proportional variance, %	29.0	19.9	16.7	—	26.7	23.3	—
Cumulative variance, %	29.0	48.9	65.6	—	26.7	50.0	—
Factor Cronbach Alpha, α	0.950	0.912	0.910	—	0.919	0.912	—
Overall, McDonald's Omega, *ω_h_*	0.960			—	0.954		—

Abbreviations: %, percentage; α, Cronbach Alpha, *ω_h_*, McDonald's Omega.

In contrast, the EFA on Italian data identified two factors (Table 2; Supplementary Figures 3 and 4). Factor 1, “Inadequate Nursing System Capacity and Alignment,” indicates that the structural and operational capacity (number, skills, roles, care models, resources, logistics) is not well aligned with patient care demands (explained variance = 26.7%). Factor 2, “Systemic Operational Strain and Breakdown in Coordination/Communication,” refers to a scenario in which the operational system—including team coordination, communication, and responsiveness—is under strain, leading to breakdowns in care delivery. This factor highlights how external pressures (such as rising complexity or volume) and internal deficiencies (equipment failure, communication gaps, collaboration failures) interact to degrade the system's ability to function cohesively (explained variance = 23.3%). The total variance explained is 50%, with an internal consistency of 0.948. In both EFAs, the complexity indicators were mostly above one (Israel: 1.066 to 2.071; Italy: 1.139 to 1.995), suggesting that items load on more than one factor, as also shown in Table 2.

The Mokken analysis (Table 3) was conducted to assess differences in the order of reasons given for UNC by CCNs. The analysis showed a good model fit (Loevinger's H coefficient > 0.50) in both data sets. In Italy, nearly all items reported H indexes ≥ 0.30, except for item 2, “Inadequate number of administrative personnel.” In the Israel data set, six items were below the threshold: 3, “Inadequate skills mix”; 5, “Unbalanced workloads”; 6, “Unbalanced nursing competencies”; 11, “Inadequate skills/competences”; 12, “Inadequate confidence in performing tasks”; and 13, “Underuse or under recognition of nursing competence.” Overall, these items should be removed from the final tool for use at the country level.

**TABLE 3:** Mokken analysis on Israel and Italian data.

	Israel							Italy						
Item	H	SE	Mon_crit	item_ord_crit.tsig	item_ord_crit.crit	item_sel	Item order	H	SE	Mon_crit	item_ord_crit.tsig	item_ord_crit.crit	item_sel	Item order
1. Inadequate number of nurses	0.340	0.042	0	3	173	1	7	0.415	0.053	0	0	52	1	1
2. Inadequate number of administrative personnel	0.370	0.050	0	3	188	1	22	0.270	0.063	0	0	18	1	18
3. Inadequate skill‐mix (e.g., excessive number of nurses without advanced education)	0.024	0.055	337	2	160	1	6	0.426	0.046	0	0	28	1	13
4. Inadequate nursing care model (e.g., functional tasks‐oriented model of care)	0.418	0.047	0	5	349	0	17	0.451	0.044	0	0	46	1	8
5. Unbalanced workloads assigned across nurses during the shift	0.398	0.038	0	3	243	1	8	0.451	0.045	0	0	0	1	6
6. Unbalanced nursing competences in the shift (e.g., excessive number of newly hired/non‐expert nurses)	0.084	0.056	297	4	375	0	1	0.434	0.048	0	0	24	1	19
7. Unexpected rise in patient acuity/complexity	0.267	0.049	0	4	233	0	10	0.422	0.047	0	0	52	1	7
8. Increased workload during the shift (e.g., increased numbers of patients at need)	0.393	0.036	0	5	245	0	4	0.460	0.049	0	0	15	1	2
9. Inaccurate initial priority setting	0.473	0.039	0	1	108	1	19	0.479	0.045	0	1	45	1	16
10. Inadequate priority re‐assessment during the shift	0.485	0.040	0	0	112	1	20	0.524	0.042	0	0	52	1	21
11. Inadequate skills/competences of nurses	0.145	0.052	215	0	39	1	5	0.503	0.041	0	0	55	1	17
12. Inadequate confidence in performing skills by nurses	0.078	0.053	286	0	76	1	2	0.522	0.042	0	3	226	0	20
13. Underuse or under recognition of the nursing competences	0.105	0.055	215	0	53	1	3	0.463	0.046	0	1	150	1	14
14. Repeated interruptions of care activities (e.g., unplanned or unexpected activities)	0.390	0.039	0	0	0	1	9	0.452	0.046	0	0	64	1	5
15. Medications prescribed not available	0.493	0.038	0	0	29	1	21	0.421	0.050	0	0	71	1	22
16. Equipment not available/not functioning properly when needed	0.502	0.039	0	0	47	1	23	0.477	0.047	0	0	63	1	23
17. Other multidisciplinary team did not provide the service expected (e.g., delays in obtaining the home advice for palliative care, mental health care)	0.435	0.038	0	0	57	1	15	0.505	0.042	0	1	96	1	10
18. Caregiver unavailable (e.g., to inform him/her about the care to be continued at home)	0.447	0.036	0	0	0	1	13	0.481	0.044	0	5	313	0	9
19. Incomplete/interrupted communication between the community nurses and the hospital nursing staff	0.457	0.037	0	0	0	1	12	0.482	0.044	0	1	112	1	4
20. Incomplete/interrupted communication among the community nursing staff	0.465	0.038	0	0	26	1	18	0.567	0.034	0	1	117	1	12
21. Incomplete/interrupted communication between nursing and medical staff	0.479	0.035	0	1	61	1	14	0.480	0.044	0	0	33	1	3
22. Lack of support/collaboration among the community team members	0.464	0.037	0	1	94	0	16	0.518	0.041	0	0	20	1	11
23. Excess time spent travelling to reach the homes or the community centers	0.398	0.043	0	0	31	1	11	0.400	0.048	0	1	74	1	15
Total scores	0.356	0.031				1		0.462	0.035				1	

Abbreviations: H, scalability index; SE, Standard Error.

### 3.3 Comparison of Unc Reasons Across Countries

The average scores differed between the Israeli and Italian CCNs (Table 4), with no significant differences in only a few items (e.g., “Unbalanced workloads assigned across nurses during the shift”). Italian CCNs identified the most important reasons for UNC as “Inadequate number of nurses” (3.14, 95% CI 2.99–3.30) and “Increased workload during the shift” (3.10, 95% CI 2.95–3.25). In contrast, Israeli CCNs considered the most important reasons for UNC to be “Unbalanced nursing competences in the shift” (3.12, 95% CI 2.92–3.31), “Inadequate confidence in nurses’ performance skills” (3.11, 95% CI 2.93–3.28), and “Underuse or under recognition of nursing competences” (3.09, 95% CI 2.90–3.28).

**TABLE 4:** Means of reasons for UNC as perceived by community nurses in Israel and Italy.

Items	Israel	Italy	*p*‐value
	Mean^*^ (CI95%)	Mean^*^ (CI95%)	
1. Inadequate number of nurses	2.78 (2.57–2.99)	3.14 (2.99–3.30)	0.044
2. Inadequate number of administrative personnel	2.07 (1.88–2.26)	2.51 (2.35–2.67)	<0.001
3. Inadequate skill‐mix (e.g., excessive number of nurses without advanced training compared to nurses with advanced training)	2.90 (2.69–3.10)	2.66 (2.49–2.84)	0.047
4. Inadequate nursing care model (e.g., functional tasks‐oriented model of care)	2.21 (2.01–2.40)	2.85 (2.70–3.02)	<0.001
5. Unbalanced workloads assigned across nurses during the shift	2.74 (2.53–2.95)	2.91 (2.74–3.09)	0.377
6. Unbalanced nursing competences in the shift (e.g., excessive number of newly hired or non‐expert nurses compared to experts)	3.12 (2.92–3.31)	2.46 (2.28–2.64)	<0.001
7. Unexpected rise in patient acuity/complexity	2.59 (2.39–2.80)	2.85 (2.70–3.01)	0.120
8. Increased workload during the shift (e.g., increased numbers of patients in need)	2.98 (2.77–3.18)	3.10 (2.95–3.25)	0.913
9. Inaccurate initial priority setting	2.15 (1.95–2.36)	2.51 (2.34–2.68)	0.005
10. Inadequate priority re‐assessment during the shift	2.10 (1.92–2.29)	2.43 (2.27–2.60)	0.005
11. Inadequate skills/competences of nurses	3.07 (2.88–3.26)	2.48 (2.30–2.65)	<0.001
12. Inadequate confidence in performing skills by nurses	3.11 (2.93–3.28)	2.43 (2.26–2.60)	<0.001
13. Underuse or under recognition of the nursing competences	3.09 (2.90–3.28)	2.64 (2.46–2.83)	<0.001
14. Repeated interruptions of care activities (e.g., unplanned or unexpected activities)	2.60 (2.39–2.81)	2.89 (2.73–3.05)	0.094
15. Medications prescribed not available	1.98 (1.77–2.18)	2.42 (2.23–2.62)	0.002
16. Equipment not available/not functioning properly when needed	2.01 (1.82–2.21)	2.37 (2.18–2.55)	0.008
17. Other multidisciplinary team did not provide the service expected (e.g., delays in obtaining the home advice for palliative care, mental health care)	2.22 (2.01–2.43)	2.76 (2.59–2.94)	<0.001
18. Caregiver unavailable (e.g., to inform him/her about the care to be continued at home)	2.36 (2.16–2.56)	2.85 (2.69–3.01)	<0.001
19. Incomplete or interrupted communication between the community nursing staff and the hospital nursing staff	2.42 (2.21–2.62)	2.91 (2.74–3.07)	0.001
20. Incomplete or interrupted communication among the community nursing staff	2.24 (2.05–2.44)	2.65 (2.47–2.83)	0.003
21. Incomplete or interrupted communication between nursing and medical staff	2.37 (2.18–2.57)	2.96 (2.79–3.13)	<0.001
22. Lack of support/collaboration among the community team members	2.29 (2.09–2.49)	2.73 (2.56–2.90)	0.001
23. Excess time spent travelling to reach homes or the community centers	2.43 (2.24–2.62)	2.57 (2.38–2.75)	0.293

Abbreviations: CI, Confidence Interval; UNC, Unfinished Nursing Care. ^*^4‐point Likert scale, from 1 “not significant reason” to 4 “very significant reason”.

Italian nurses perceived the following reason as less significant: “Equipment not available/not functioning properly when needed” (2.37, 95% CI 2.18–2.55) while Israeli nurses considered “Medications prescribed not available” (1.98, 95% CI 1.77–2.18) as less influential.

## 4 Discussion

The study aimed to develop and validate a tool for measuring reasons for UNC in community care, a context in which all countries are increasing investment, especially after the pandemic revealed a lack of care outside hospitals (Polin et al. 2021; Ruzafa‐Martinez et al. 2023). Our aim was to accelerate the generation of evidence to support the expanding provision of community‐based care (Polin et al. 2021; Ruzafa‐Martinez et al. 2023; Palese 2026). For this reason, we selected a well‐established tool, and further developed its structure simultaneously in two countries.

### 4.1 Participants

Approximately 300 CCNs participated, with half from each country. On average, each CN provides care to about 3000 people in the community (e.g., Table S2), so it can be estimated that participants were responsible for communities of approximately 450,000 people per country. The participating CCNs were mainly female and senior experts in terms of experience. In Israel, almost half had been educated at the postgraduate level (Drach‐Zahavy and Srulovici 2019), while in Italy they had completed a nursing degree and some postgraduate courses. Working in the community is complex, and despite several interactions with other professionals (general practitioners, social services), the role requires CCNs to work mainly independently or in isolation. Therefore, strong nursing experience, accompanied by advanced education, may help when independent decisions are required. In both countries, nurses are required to work extra hours, with changes in shifts due to absences, suggesting similar organizational instability that requires continuously evolving strategies to provide the expected care. While the level of nursing resources was mostly perceived as adequate, in Italy a lack of nursing aides has emerged. In this country, the profile of nursing aides has been long established and more recently redesigned (A Brugnolli et al. 2024): the perceived shortage suggests that the community healthcare system requires reorganization in terms of support resources and skill mix capable of supporting CNN.

### 4.2 Unfinished Community Nursing Care Reasons Survey

The UCNC_RS tool was validated for content and language and comprised 23 items. However, the EFA conducted on Israeli data identified three factors, while the analysis of Italian data revealed two factors with several cross‐loading likelihood because the limited sample power. In both countries, nurses reported two main categories of UNC reasons: (a) system operational issues leading to poor coordination, breakdowns, and workflow problems (Israel: Factor 1, “Care Coordination and Resource Availability Challenges,” Factor 2, “Challenges in Nursing Workflow and Care Prioritization”; Italy: Factor 2, “Systemic Operational Strain and Breakdown in Coordination/Communication”); and (b) issues related to specific nursing capacity (Israel: Factor 3, “Nursing Skill Mix and Competence Alignment”; Italy: Factor 1, “Inadequate Nursing System Capacity and Alignment”).

Despite empirical differences, the factor structures identified in the two national datasets showed notable consistency in the categories of reasons for UNC. Specifically, the observed cross‐loading patterns suggest an underlying convergence around two broad domains: team relationships and functioning, and resource‐related issues. Additionally, the distinction found in the Israeli data between the availability and competence of resources points to potentially meaningful subdimensions within the resource domain. These findings indicate that alternative factor solutions—such as imposing two‐ or three‐factor models could provide further theoretical and empirical insights. However, at this early stage of instrument development and validation, we deliberately chose not to impose alternative or forced factor structures. This decision was guided by the exploratory nature of the study, the absence of a predefined theoretical framework, and the high contextual complexity of community care systems (European Commission 2018). Community care is strongly influenced by national organization, professional roles, and local service configurations; therefore, we aimed to respect these contextual specificities by allowing factor structures to emerge independently within each dataset rather than constraining them with cross‐country analytical assumptions. Moreover, forcing factor solutions at this stage could obscure meaningful contextual differences in how UNC is conceptualized and experienced across settings. Preserving these differences was especially important in this exploratory phase, where the goal is to validate a tool rather than prematurely harmonize it. Nevertheless, the observed convergence across datasets provides important direction for future research. The distinction between resources and team functioning, as well as between resource availability and competence, offers a promising foundation for theory‐driven refinement of the instrument. Future studies should build on these findings by testing forced factor solutions, applying alternative rotation methods, and conducting confirmatory factor analyses in larger and more diverse samples. Such work will allow the integration of emerging theoretical insights with empirical evidence, ultimately strengthening the structural validity of the tool.

Overall, the findings suggest that the underlying reasons differ between countries, with varying capacities to explain the variance of UNC in community care. In contrast, accumulated evidence from hospital settings (e.g., Antoszewska and Gutysz‐Wojnicka 2025; Palese et al. 2021) has revealed consistent and well‐defined patterns of reasons for UNC worldwide. Furthermore, analysis of the complexity indexes shows that items fail in both countries on more than one factor, indicating that the reasons for UNC in community care operate differently from those identified in hospitals. This may be related to greater standardization of workflows, including nursing processes, within hospitals. In community settings, the variability of patient needs, interpersonal dynamics, contextual factors, and the multilevel nature of activities—ranging from prevention to palliative care—may lead to more diverse and context‐dependent reasons for UNC. Therefore, the complexity of community nursing care (Jackson et al. 2015) seems to prevent the existence of a clear set of common UNC factors; moreover, the multiple functions performed by CCNs, from healthcare promotion to disease management (European Commission 2018; Dellafiore et al. 2022; Zeydani et al. 2023), may require different resources, and consequently, the incidence of UNC may be due to a range of reasons that cannot be integrated into a multidimensional construct. In addition, reasons can be conceptualized within a systems‐level context, where the intersection of political, professional, and organizational factors impacts the provision of care at the community level (Phelan et al. 2018b). As a result, nurses may not be able to perceive this complexity in daily care, although they may recognize each influencing element and its sequence. This explanation is supported by the Mokken analysis findings, where (a) not all items reported good H indexes, and (b) items show a different rank order according to their H values in each country. The fact that not all items have been retained suggests that not all of them influence UNC. Specifically, in the Israeli context, compared to the Italian one, all items related to nurses’ competencies (e.g., inadequate skill mix; unbalanced nursing competencies in shift skills) should be removed. This may be due to the advanced education level of Israeli nurses and the maturity of the model, which in Italy was only formally recognized in 2022 (Fanton et al. 2026).

The differing order of items suggests that their influence on the UNC phenomenon occurs at different times: while workloads (the nurse‐to‐patient ratio) were the primary factor for Italian CCNs, unbalanced competencies during shifts and a lack of confidence emerged among Israeli nurses. Italian nurses cited reasons similar to those already reported by hospital nurses, such as staff shortages (e.g., Palese et al. 2015), inability to manage shifts due to increased workload (e.g., Blackman et al. 2018), and inadequate communication between nurses and doctors (e.g., Blackman et al. 2020; Chiappinotto and Palese 2022). In contrast, the factors perceived as most influential by Israeli nurses all relate to the inadequacy of community nurses' competencies. This difference can be explained by the distinct two‐tier health systems in Italy and Israel. Hospital bed rates in Israel and Italy reflect significant variations in healthcare approaches and resource allocation: Israel has a low hospital bed rate (2.91 per 1000 citizens) compared to Italy (3.09) and the Organization for Economic Co‐operation and Development average (4.4), along with a much shorter average length of stay (4.6 days in Israel vs. 7.4 days in Italy). This shorter average length of stay in Israel, combined with fewer hospital beds, suggests a healthcare system that prioritizes efficient use of hospital resources and may indicate a stronger emphasis on outpatient and community‐based health services. As a result, Israeli community nurses are required to manage more complex medical conditions that are typically addressed in hospital settings in Italy. Israel's strategy aligns with its Ministry of Health's promotion of home hospitalization and rehabilitation services, while Italy has only recently implemented a national policy for the development of community nursing care. These systemic differences not only shape the respective healthcare landscapes but also significantly influence the scope and complexity of community nursing practices (Jones et al. 2020), with Israeli nurses assuming greater responsibility for post‐acute care management in the community setting.

At the macro level (Jones et al. 2019), Italian nurses primarily identified systemic issues such as staff shortages and high workloads as the main causes of UNC, indicating structural challenges in staffing and healthcare resource allocation. In contrast, Israeli nurses focused less on staffing numbers and more on the underuse and under‐recognition of nursing skills, suggesting a system where nurses may be present but are not fully empowered or supported. At the meso level (Jones et al. 2019), both groups reported similar organizational issues, including unbalanced workloads and care interruptions during shifts. However, Italian nurses also emphasized poor communication with medical staff as a key barrier, reflecting possible coordination problems within teams. Finally, at the micro level (Jones et al. 2019), Italian nurses were less likely to view individual skills or confidence as major issues, focusing instead on external factors. Israeli nurses, however, considered nurses’ confidence and competence central to explaining UNC, suggesting a more self‐critical or skill‐focused professional culture.

### 4.3 Limitations

The study has several limitations. First, during the various phases of the validation process, nurses were primarily involved as participants; however, future studies should include additional stakeholders, given the complexity of care processes in community settings, which involve multiple actors and systems. Second, content validity was assessed through several meetings with the international research group, resulting in a final consensus on the included items, but specific indices such as the Content Validity Index were not calculated. Although respondents from different countries may interpret some items differently, leading to different factor structures, further development of evidence for structural validity is recommended. We also conducted a separate factor analysis for the two countries (Israel and Italy). Given the limitations in the sample size of each group, further studies may achieve factorial solutions with larger samples. Third, reasons for UNC occurrence were assessed based on the perceptions of the CCNs; further studies may explore additional reasons for UNC in more depth, such as organizational factors as the nurse manager's leadership style (Srulovici and Yanovich 2022), nurse‐related factors such as personal accountability (Srulovici and Drach‐Zahavy 2017), and patient‐related factors such as the complexity of their needs (Abdelhadi et al. 2023).

## 5 Conclusions

Our aims were to develop and validate a tool and to compare the reasons across contexts through a methodological study conducted simultaneously in two countries.

First, the UCNC_RS comprises 23 items representing different reasons for UNC, organized hierarchically. Attenuating one element may influence the next, suggesting that interventions to prevent or mitigate UNC can be ordered as a de‐escalation strategy. Overall, the UCNC_RS can be used in daily practice to survey and proactively address factors that may increase the occurrence of UNC.

Second, the reasons for UNC differ between countries, both in their order of importance and in their scores, with only some common elements, indicating different patterns of perceived reasons. Unlike in hospitals, measuring reasons for UNC in community care is challenging; continuing to investigate investigation the nature and frequency of these reasons may help detect changes and inform targeted policies.

## Author contributions

**Stefania Chiappinotto, Einav Srulovici, Anat Drach‐Zahavy, Alvisa Palese**: conceptualization. **Luca Grassetti, Alvisa Palese**: formal analysis. **Stefania Chiappinotto, Galit Palacios Klein, Nihal Khatib, Aysun Bayram**: investigation, Stefania Chiappinotto, Luca Grassetti, Alvisa Palese, Aysun Bayram: Validation. **Stefania Chiappinotto, Einav Srulovici, Galit Palacios Klein, Nihal Khatib, Anat Drach‐Zahavy, Alvisa Palese, Aysun Bayram**: project administration. **Stefania Chiappinotto, Einav Srulovici, Galit Palacios Klein, Nihal Khatib, Luca Grassetti, Anat Drach‐Zahavy, Alvisa Palese, Aysun Bayram**: writing—review and editing. **Luca Grassetti, Alvisa Palese: Methodology. Einav Srulovici, Anat Drach‐Zahavy, Alvisa Palese**: supervision. **Luca Grassetti, Alvisa Palese, Aysun Bayram, Stefania Chiappinotto**: data curation. **Stefania Chiappinotto, Alvisa Palese, Luca Grassetti**: writing—original draft.

## Funding

This research did not receive any specific grant from funding agencies in the public, commercial, or not‐for‐profit sectors.

## Conflicts of Interest

The authors declare no conflicts of interest.

## Supporting information




**Supporting Information 1**: phn70140‐sup‐0001‐Figure1.docx.

**Supporting Information 2**: phn70140‐sup‐0002‐Figure2.docx.

**Supporting Information 3**: phn70140‐sup‐0003‐Figure3.docx.

**Supporting Information 4**: phn70140‐sup‐0004‐Figure4.docx.

**Supporting Information 5**: phn70140‐sup‐0005‐Table1.docx.

**Supporting Information 6**: phn70140‐sup‐0006‐Table2.docx.

**Supporting Information 7**: phn70140‐sup‐0007‐Table3.docx.

**Supporting Information 8**: phn70140‐sup‐0008‐Table4.docx.

## Data Availability

The data that support the findings of this study are available from the corresponding author upon reasonable request.
