# Supplementary material for: Measuring Reasons for Unfinished Nursing Care as Perceived by Community Care Nurses: A Binational Development and Validation Study
Source: Public Health Nurs. 2026 Jun 3;43(5):1394–408. doi: 10.1111/phn.70140 (PMC13535899; doi:10.1111/phn.70140)
Supplement: Supplementary file 1 [file PHN-43-1394-s001.docx]

**Supplementary Table 2.** The Community Care Nurses profile role in Israel and Italy: a brief description.

***Israel***: Community Care Nurses exists in Israel since the early 20th century. The nursing profession is regulated by existing regulations and ordinances, but not by a specific nursing law. Nursing practice in Israel is governed by the Public Health Regulations of 1981 (nursing staff in clinics) and 1988 (nursing staff in hospitals). The role of a community nurse is broadly defined and includes health promotion, monitoring chronic patients, providing treatments in clinics, home care, and promoting quality metrics (Nissanholtz-Gannot, Rosen & Hirschfeld, 2017). Community Nurses perform a variety of tasks and activities, such as conducting medical tests, treating chronic patients with heart failure, diabetes, hypertension, and oncological conditions; monitoring these patients, planning interventions and follow-ups, making home visits, and handling administrative tasks such as reviewing hospital discharge summaries. Their responsibilities also include pharmacological interventions, wound care management, immunization services, and monitoring vital signs (Ministry of Health, 1988).

***Italy***: The Community and Family Care Nursing role was formally established by the Decree No. 77/2022 (Presidente della Repubblica, 2020). However, Community and Family Care Nurses had already been introduced in various regions through individual initiatives (e.g., Giunta Regionale Friuli Venezia Giulia, 2022) that inspired national policies. In line with international guidelines (European Commission, 2018), they play a central role in community care, planning and delivering care based on identified needs, and collaborating with health care professionals to ensure interdisciplinary integration of health and social services. Community nurses are also responsible for identifying emerging health problems, delivering proactive health care management, promotion, and prevention, and involving both families and the community in care processes while integrating different care settings (Ministero della Salute, 2022a). The decree established a standard of one Community and Family Care Nurse for every 3,000 citizens (Ministero della Salute, 2022b).

European Commission. (2018). *Report dell’Expert Panel on effective ways of investing in Health (EXPH), Tools and Methodologies for Assessing the Performance of Primary Care.* https://health.ec.europa.eu/document/download/c3e2c0c3-18cd-4155-8339-4456d619e0f7_en

Giunta Regionale Friuli Venezia Giulia. (2022). *L’infermiere di Famiglia o Comunità: il modello assistenziale in Friuli Venezia Giulia.* <https://asufc.sanita.fvg.it/export/sites/asufc/it/documenti/inf_comunita-policy_regionale.pdf>

Ministero della Salute. (2022a). *Regolamento recante la definizione di modelli e standard per lo sviluppo dell’assistenza territoriale nel Servizio sanitario nazionale.* <https://www.quotidianosanita.it/allegati/allegato1655970392.pdf>

Ministero della Salute. (2022b). *Regolamento recante la definizione di modelli e standard per lo sviluppo dell’assistenza territoriale nel Servizio sanitario nazionale.* <https://www.google.com/url?sa=t&source=web&rct=j&opi=89978449&url=https://www.quotidianosanita.it/allegati/allegato1655970392.pdf&ved=2ahUKEwjJuePE6I2QAxVVhP0HHTIoOR8QFnoECDwQAQ&usg=AOvVaw3MfyodocaA3S3mll-DMRXV>

Ministry of Health. (1988). *The Public Health Regulations (Nursing Practitioners in Hospitals).* <https://www.nevo.co.il/law_html/law00/74747.htm>

Nissanholtz-Gannot, R., Rosen, B., Hirschfeld, M., & Community Nursing Study Group (2017). The changing roles of community nurses: the case of health plan nurses in Israel. *Israel journal of health policy research*, *6*(1), 69. https://doi.org/10.1186/s13584-017-0197-5

Presidente della Repubblica (2020). *Misure urgenti in materia di salute, sostegno al lavoro e all'economia, nonché di politiche sociali connesse all'emergenza epidemiologica da COVID-19.* https://www.gazzettaufficiale.it/eli/id/2020/07/18/20A03914/sg
