# Supplementary material for: Measuring Reasons for Unfinished Nursing Care as Perceived by Community Care Nurses: A Binational Development and Validation Study
Source: Public Health Nurs. 2026 Jun 3;43(5):1394–408. doi: 10.1111/phn.70140 (PMC13535899; doi:10.1111/phn.70140)
Supplement: Supplementary file 3 [file PHN-43-1394-s003.docx]

**Supplementary Table 3.** Unfinished Nursing Care Survey (Bassi et al., 2020) tool adaptation and development

| **UNCS reasons (n=18)** | **UCNC_RS tool**  **Decisions regarding item inclusion or exclusion and their adaptation** |
| --- | --- |
| Tension/conflicts within the nursing staff | *Deleted*  Perceived as redundant to “Incomplete or interrupted communication among nursing staff” |
| Incomplete or interrupted communication among nursing staff | *Reworded*  Incomplete or interrupted communication among the community nursing staff |
| Tension/conflicts between nursing and medical staff | *Deleted*  Perceived as redundant to “Incomplete or interrupted communication between nursing and medical staff” |
| Incomplete or interrupted communication between nursing and medical staff | *Unchanged*  Incomplete or interrupted communication between nursing and medical staff |
| Lack of support/collaboration among team members | *Reworded*  Lack of support/collaboration among the community team members |
| Inadequate nursing care model (e.g., functional tasks-oriented model of care) | *Unchanged*  Inadequate nursing care model (e.g., functional tasks-oriented model of care) |
| Inaccurate initial priority setting | *Unchanged*  Inaccurate initial priority setting |
| Inadequate priority reassessment during the shift | *Unchanged*  Inadequate priority re-assessment during the shift |
| Nurse aides missed or delayed the reporting of the tasks left undone | *Deleted*  Because no nurse aides are present in the considered settings |
| Inadequate supervision of the tasks assigned to the nurse aides | *Deleted*  Because no nurse aides are present in the considered settings |
| Incomplete or interrupted communication between nursing staff and nurse aides/assistive personnel | *Deleted*  Because no nurse aides are present in the considered settings |
| Medications prescribed not available | *Unchanged*  Medications prescribed not available |
| Equipment not available/not functioning properly when needed | *Unchanged*  Equipment not available/not functioning properly when needed |
| Other departments did not provide the service expected (e.g., delay in diagnostic processes) | *Reworded*  Other multidisciplinary team did not provide the expected service (e.g., delays in obtaining home advice for palliative care or mental health care) |
| Inadequate number of nurses | *Unchanged*  Inadequate number of nurses |
| Inadequate number of staff support | *Deleted*  Because no nurse aides are present in the considered settings |
| Unexpected increase in the number of patients in critical conditions | *Reworded*  Unexpected increase in patient acuity or complexity |
| High number of hospitalisations/discharges during the shift | *Deleted*  Perceived as not pertinent to the community setting |
|  | *Added*  Inadequate number of administrative personnel  Inadequate skill mix (e.g., excessive number of nurses without advanced training compared to nurses with advanced training)  Unbalanced workloads assigned across nurses  Unbalanced nursing competencies during the shift (e.g., excessive number of newly hired or non-expert nurses compared to experts)  Increased workload during the shift (e.g., increased number of patients in need)  Inadequate skills or competencies of nurses  Inadequate confidence in performing skills by nurses  Underuse or underrecognition of nursing competencies  Repeated interruptions of care activities (e.g., unplanned or unexpected activities)  Caregiver unavailable (e.g., to inform him or her about the care to be continued at home)  Incomplete or interrupted communication between community nursing staff and hospital nursing staff  Excess time spent traveling to reach homes or community centers |

**Legend:** UCNC_RS, Unfinished Community Nursing Care Reasons Survey; UNCS, Unfinished Nursing Care Survey.
