# Supplementary material for: Measuring Reasons for Unfinished Nursing Care as Perceived by Community Care Nurses: A Binational Development and Validation Study
Source: Public Health Nurs. 2026 Jun 3;43(5):1394–408. doi: 10.1111/phn.70140 (PMC13535899; doi:10.1111/phn.70140)
Supplement: Supplementary file 4 [file PHN-43-1394-s004.docx]

**Supplementary Figure 2**. Correlation plot on Israeli data

**Legend:** Reasons and their corresponding numbers refer to the items of the Unfinished Community Care Nurse *Reason* tool. Values indicate the correlations between items, ranging from –1 to +1, with negative and positive signs reflecting the direction of the relationship.
