# Supplementary material for: Measuring Reasons for Unfinished Nursing Care as Perceived by Community Care Nurses: A Binational Development and Validation Study
Source: Public Health Nurs. 2026 Jun 3;43(5):1394–408. doi: 10.1111/phn.70140 (PMC13535899; doi:10.1111/phn.70140)
Supplement: Supplementary file 5 [file PHN-43-1394-s005.docx]

**Supplementary Table 1.** COnsensus-based Standards for the selection of health Measurement INstruments (COSMIN) guidelines (Mokkink et al., 2018)

In our study, the concept of PROM as Patient Reported Outcome measures was considered as the perception of Community Care Nurses regarding the Unfinished Nursing Care reasons.

| **Item** | **Gold standard** | **In the study** |
| --- | --- | --- |
| ***General recommendations for the design of a study on measurement properties*** | | |
| *Research aim* | | |
| 1. Provide a clear research aim, including (1) the name and version of the PROM, (2) the target population, and (3) the measurement properties of interest | Research aims clearly described | See Aims – end of the background |
| *PROM* | | |
| 2. Provide a clear description of the construct to be measured | Construct clearly described | See Background  See Study phases |
| 3. Provide a clear description of the development process of the PROM, including a description of the target population for which the PROM was developed | Development process clearly described | See Study phases |
| 4. The origin of the construct should be clear: provide a theory, conceptual framework (i.e., reflective or formative model) or disease model used or clear rationale to define the construct to be measured | Origin of the construct clear | See Study phases |
| 5. Provide a clear description of the structure of the PROM (i.e., the number of items and subscales included in the PROM, instructions given and response options) and its scoring algorithm | Structure and scoring algorithm clearly described | See Study phases |
| 6. Provide a clear description of existing evidence on the quality of the PROM | Existing evidence on the quality of the PROM clearly described | See Study phases |
| 7. Provide a clear description of the context of use* | Context of use clearly described | See Design and setting |
| *Target population* | | |
| 8. Provide a clear description of in- and exclusion criteria to select patients, e.g., in terms of disease condition and characteristics like age, gender, language or country, and setting (e.g., general population, primary care or hospital/rehabilitation care) | In- and exclusion criteria for patients clearly described | See Study phases |
| 9. Provide a clear description of the method used to select the patients for the study (e.g., convenience, consecutive, or random) | Method for patient selection clearly described | See Study phases |
| 10. Describe whether the selected sample represents the target population in which the PROM will be used in terms of age, gender, important disease characteristics (e.g., severity, status, duration) | Study sample representing the target population clearly described | See Study phases |
| ***Content validity*** | | |
| *Design requirements* | | |
| 1. From the perspective of the patients: use an appropriate method for assessing (1) the relevance of each item for the patients’ experience with the condition, AND (2) the comprehensiveness of the PROM, AND (3) the comprehensibility of the PROM instructions, items, response options, and recall period | Widely recognized or well justified methods for qualitative research will be used to assess the three aspects | NA |
| 2. From the perspective of professionals: use an appropriate method for assessing (1) the relevance of each item for the construct of interest, AND (2) the comprehensiveness of the PROM | Widely recognized or well justified methods for qualitative research will be used to assess the two aspects | See Study phases  See Supplementary Table 2 |
| 3. Include professionals from all relevant disciplines | Professionals from all required disciplines will be included | See Study phases |
| 4. Evaluate each item in an appropriate number of patients or professionals   For qualitative studies  For quantitative (survey) studies | ≥7  ≥50 | See Study phases |
| 5. Use skilled group moderators or interviewers | Skilled group moderators or interviewers will be used | See Study phases |
| 6. Base the group meetings or interviews on an appropriate topic or interview guide | Appropriate topic or interview guide will be used | See Study phases |
| 7. Record and transcribe verbatim the group meetings or interviews | All group meetings or interviews will be recorded and transcribed verbatim | See Study phases |
| *Analyses* | | |
| 8. Use an appropriate approach to analyse the data | A widely recognized or well justified approach will be used | See Study phases |
| 9. Involve at least two researchers in the analysis | At least two researchers will be involved in the analysis | See Study phases |
| ***Structural validity*** | | |
| *Statistical methods* | | |
| 1. For CTT: perform confirmatory factor analysis | Confirmatory factor analysis will be performed | NA |
| 2. For CTT: provide clear information on how the factor analysis will be performed, e.g., software program, method of estimation, whether and how assumptions will be checked, rotation method, criteria for model fit. | Clear information on the performance of the analysis is provided | See Study phases |
| 3. For IRT/Rasch: choose a model that fits to the research question | Chosen model fits well to the research question | See Study phases |
| 4. For IRT/Rasch: provide clear information on how the IRT or Rasch analysis will be performed, e.g., software program, which IRT or Rasch model used, method of estimation, whether and how assumptions will be checked, criteria for model fit. | Clear information on the performance of the analysis is described | See Study phases |
| 5. Perform the analysis in a sample with an appropriate number of patients (considering expected number of missing values) | FA: 7 times number of items and ≥100  Rasch/1PL models: ≥ 200 patients  2PL parametric IRT models OR Mokken scale analysis: ≥ 1000 patients | See Study phases |
| 6. Provide a clear description of how missing items will be handled | The way missing items will be handled is clearly described | See Study phases |
| ***Internal consistency*** | | |
| *Design requirements* | | |
| 1. Check whether a scale or a subscale is unidimensional | Evidence provided that each scale or subscale is unidimensional | See Study phases |
| 2. Perform the analysis in a sample with an appropriate number of patients (taking into account expected number of missing values) | ≥100 patients | See Study phases |
| 3. Provide a clear description of how missing items will be handled | The way missing items will be handled is clearly described | See Study phases |
| *Statistical methods* | | |
| 4. For continuous scores: calculate Cronbach’s alpha or Omega for each unidimensional scale or subscale | Cronbach’s alpha, or Omega will be calculated | See Study phases |
| 5. For dichotomous scores: calculate Cronbach’s alpha or KR-20 for each unidimensional scale or subscale | Cronbach’s alpha or KR-20 will be calculated | NA |
| 6. For IRT-based scores: calculate standard error of theta (SE (θ)) or reliability coefficient of estimated latent trait value (index of (subject or item) separation) for each unidimensional scale or subscale | SE(θ) or reliability coefficient will be calculated | See Study phases |
| ***Cross-cultural validity\Measurement invariance*** | | |
| *Design requirements* | | |
| 1. Provide a clear description of the group variable(s), including dichotomization or categorization | The group variable(s) is/are clearly described | See Design and setting |
| 2. Provide a clear description of the relevant characteristics of the patients that should be similar in both subgroups, such as demographic or disease characteristics | Relevant characteristics are clearly described | See Design and setting |
| 3. Perform the analysis in a sample with an appropriate number of patients (considering expected number of missing values) | Regression analyses or IRT/Rasch based analyses: ≥200 patients per group  OR  MGCFA*: 7 times number of items and ≥100 patients | NA |
| *Statistical methods* | | |
| 4. For CTT: perform a multi-group confirmatory factor analysis (MGCFA) | MGCFA will be performed | See Study phases |
| 5. For CTT: provide clear information on how the factor analysis will be performed, e.g., software program, method of estimation, criteria for model fit, and whether and how assumptions were checked | Clear information on the performance of the analysis is provided | See Study phases |
| 6. For IRT/Rasch: perform differential item functioning (DIF) analyses | DIF will be performed | See Study phases |
| 7. For IRT/Rasch: provide clear information on how the IRT or Rasch analysis will be performed, e.g., software program, which IRT or Rasch model used, method of estimation, criteria for model fit, whether and how assumptions were checked | Clear information on the performance of the analysis is provided | See Study phases |
| 8. Provide a clear description of how missing items will be handled | The way missing items will be handled is clearly described | See Study phases |
| ***Measurement error and reliability*** | | |
| *Design requirements* | | |
| 1. Use at least two measurements | At least two measurements | NA |
| 2. Ensure that the administrations will be independent | Independent measurements | NA |
| 3. Ensure that the patients will be stable in the interim period on the construct to be measured | Patients will be stable (evidence provided) | NA |
| 4. Use an appropriate time interval between the two measurements, which is long enough to prevent recall, and short enough to ensure that patients remain stable | Time interval appropriate | NA |
| 5. Ensure that the test conditions will be similar for the measurements (e.g., type of administration, environment, instructions) | Test conditions similar (evidence provided) | NA |
| 6. Perform the analysis in a sample with an appropriate number of patients (considering expected number of missing values) | ≥100 patients | NA |
| *Statistical methods for measuring error* | | |
| 7. For continuous scores: calculate the Standard Error of Measurement (SEM), Smallest Detectable Change (SDC) or Limits of Agreement (LoA) | SEM, SDC, or LoA will be calculated, and model or formula is clearly described** | NA |
| 8. For dichotomous/nominal/ordinal scores: calculate the percentage (positive and negative) agreement | % positive and negative agreement will be calculated | NA |
| 9. Provide a clear description of how missing items will be handled | The way missing items will be handled is clearly described | NA |
| *Statistical methods for reliability* | | |
| 7. For continuous scores: calculate an intraclass correlation coefficient (ICC) | ICC will be calculated, and model or formula of the ICC is clearly described** | NA |
| 8. For dichotomous/nominal/ordinal scores: calculate kappa | Kappa will be calculated | NA |
| 9. For ordinal scores: calculate a weighted kappa | Weighted Kappa calculated and weighting scheme is described | NA |
| 10. Provide a clear description of how missing items will be handled | The way missing items will be handled is clearly described | NA |
| ***Criterion validity*** | | |
| *Design requirements* | | |
| 1. Describe whether the proposed criterion can be considered as a reasonable ‘gold standard’ | Criterion can be considered an adequate ‘gold standard’ (evidence provided) | NA |
| 2. Perform the analysis in a sample with an appropriate number of patients (taking into account expected number of missing values) | ≥ 50 patients in the smallest group | NA |
| 3. Use an appropriate time schedule for assessments of the PROM of interest and ‘gold standard’ | PROM and gold standard will be administered at the same time | NA |
| *Statistical methods* | | |
| 4. For continuous scores: calculate correlations, or the area under the receiver operating curve | Correlations or AUC will be calculated | NA |
| 5. For dichotomous scores: determine sensitivity and specificity | Sensitivity and specificity will be determined | NA |
| 6. Provide a clear description of how missing items will be handled | The way missing items will be handled is clearly described | NA |
| ***Hypotheses testing for construct validity*** | | |
| ***A. Comparison with other outcome measurement instruments (convergent validity)*** | | |
| *Design requirements* | | |
| 1. Formulate hypotheses about expected relationships between the PROM under study and other outcome measurement instrument(s) | Hypotheses formulated including the expected direction and magnitude of the correlations stated | NA |
| 2. Provide a clear description of the construct(s) measured by the comparator instrument(s) | Construct(s) measured by the comparator instrument(s) is/are clearly described | NA |
| 3. Use comparator instrument(s) with sufficient measurement properties | Sufficient measurement properties of the comparator instrument(s) in a population similar to the study population | NA |
| 4. Perform the analysis in a sample with an appropriate number of patients (taking into account expected number of missing values) | ≥100 patients | NA |
| 5. Use an appropriate time schedule for assessments of the PROM of interest and comparison instruments | PROM and comparison instrument(s) will be administered at the same time | NA |
| *Statistical methods* | | |
| 6. Use statistical methods that are appropriate for the hypotheses to be tested | Statistical methods will be appropriate | NA |
| 7. Provide a clear description of how missing items will be handled | The way missing items will be handled is clearly described | NA |
| ***B. Comparison between subgroups (discriminative or known-groups validity)*** | | |
| *Design requirements* | | |
| 1. Formulate hypotheses regarding mean differences between subgroups | Hypotheses formulated including the expected directions and magnitude of the mean differences stated | NA |
| 2. Provide an adequate description of important characteristics of the subgroups, such as disease or demographic characteristics | Adequate description of the important characteristics of the subgroups | NA |
| 3. Perform the analysis in a sample with an appropriate number of patients (taking into account expected number of missing values) | ≥100 patients per group | NA |
| *Statistical methods* | | |
| 4. Use statistical methods that are appropriate for the hypotheses to be tested | Statistical methods will be appropriate | NA |
| 5. Provide a clear description of how missing items will be handled | The way missing items will be handled is clearly described | NA |
| ***Responsiveness*** | | |
| ***A. Criterion approach (i.e., comparison to a ‘gold standard’)*** | | |
| *Design requirement* | | |
| 1. The proposed criterion can be considered as a reasonable ‘gold standard’ | Criterion can be considered an adequate ‘gold standard’ (evidence provided) | NA |
| 2. Use an appropriate time schedule for assessments of the PROM of interest and the gold standard | PROM and gold standard will be administered at the same time at all occasions | NA |
| 3. Use an appropriate time interval between first and second measurements | Time interval will be appropriate | NA |
| 4. Describe anything likely to occur in the interim period (e.g., intervention, in case of progressive disease, other relevant events) | Anything likely to occur during the interim period (e.g., treatment) is adequately described | NA |
| 5. Ensure that a proportion of the patients is likely to change (i.e., improvement or deterioration) on the construct to be measured | Part of the patients is likely to change (evidence provided) | NA |
| 6. Perform the analysis in a sample with an appropriate number of patients (taking into account expected number of missing values) | ≥50 patients in the smallest group | NA |
| *Statistical methods* | | |
| 7. For continuous scores: calculate correlations between change scores, or the area under the Receiver Operator Curve (ROC) curve (AUC) | Correlations or AUC will be calculated | NA |
| 8. For dichotomous scales: calculate sensitivity and specificity (changed versus not changed) | Sensitivity and specificity will be calculated | NA |
| 9. Provide a clear description of how missing items will be handled | The way missing items will be handled is clearly described | NA |
| ***B. Construct approach (i.e., hypotheses testing; comparison with other outcome measurement instruments)*** | | |
| *Design requirements* | | |
| 1. Formulate hypotheses about expected relationships between the change scores on the PROM under study and (change scores on) other outcome measurement instrument(s) | Hypotheses will be formulated including the expected direction and magnitude of the correlations stated | NA |
| 2. Provide a clear description of the construct(s) measured by the comparator instrument(s) | Constructs measured by the comparator instrument(s) is/are clearly described | NA |
| 3. Provide information that the measurement properties of the comparator instrument(s) are sufficient | Sufficient measurement properties of the comparator instrument(s) in a population similar to the study population | NA |
| 4. Use an appropriate time schedule for assessments of PROM of interest and comparison instruments | PROM and comparison instrument will be administered at the same time at all occasions | NA |
| 5. Use an appropriate time interval between first and second measurements | Time interval appropriate | NA |
| 6. Describe anything likely to occur in the interim period (e.g., intervention, other relevant events) | Anything likely to occur during the interim period (e.g., treatment) is adequately described | NA |
| 7. Ensure that a proportion of the patients is likely to change (i.e., improvement or deterioration) on the construct to be measured | Part of the patients is likely to change (evidence provided) | NA |
| 8. Perform the analysis in a sample with an appropriate number of patients (taking into account expected number of missing values) | ≥100 patients | NA |
| *Statistical methods* | | |
| 9. Ensure that the statistical methods are adequate for the hypotheses to be tested | Statistical methods are appropriate | NA |
| 10. Provide a clear description of how missing items will be handled | The way missing items will be handled is clearly described | NA |
| ***C. Construct approach: (i.e., hypotheses testing: comparison between subgroups)*** | | |
| *Design requirements* | | |
| 1. Formulate hypotheses regarding differences between change scores of subgroups a priori (i.e., before data collection) | Hypotheses formulated including the expected differences between change scores stated | NA |
| 2. Provide an adequate description about important characteristics of the subgroups, such as disease or demographic characteristics | Adequate description of the important characteristics of the subgroups | NA |
| 3. Use an appropriate time interval between first and second administration of the measurement | Time interval appropriate | NA |
| 4. Describe anything likely to occur in the interim period (e.g., intervention, progressive disease, other relevant events) | Anything likely to occur during the interim period (e.g., treatment) is adequately described | NA |
| 5. Ensure that a proportion of the patients is likely to change (i.e., improvement or deterioration) on the construct to be measured | Part of the patients is likely to change (evidence provided) | NA |
| 6. Perform the analysis in a sample with an appropriate number of patients (taking into account expected number of missing values) | ≥100 patients | NA |
| *Statistical methods* | | |
| 7. Ensure that the statistical methods are adequate for the hypotheses to be tested | Statistical methods are appropriate | NA |
| 8. Provide a clear description of how missing items will be handled | The way missing items will be handled is clearly described | NA |
| ***D. Construct approach: (i.e., hypotheses testing: before and after intervention)*** | | |
| *Design requirements* | | |
| 1. Formulate challenging hypotheses regarding expected changes before and after intervention a priori (i.e., before data collection) | Hypotheses formulated including the expected changes stated | NA |
| 2. Provide an adequate description of the intervention to allow replication, including how and when they will be administered | Adequate description of the intervention | NA |
| 3. Use an appropriate time interval between first and second administration | Time interval appropriate | NA |
| 4. Describe anything likely to occur in the interim period (e.g., intervention, progressive disease, other relevant events) is adequately | Anything likely to occur during the interim period (e.g., treatment) is adequately described | NA |
| 5. Ensure that a proportion of the patients is likely to change (i.e., improvement or deterioration) on the construct to be measured | Part of the patients is likely to change (evidence provided) | NA |
| 6. Perform the analysis in a sample with an appropriate number of patients (taking into account expected number of missing values) | ≥100 patients | NA |
| *Statistical methods* | | |
| 7. Ensure that the statistical methods are adequate for the hypotheses to be tested | Statistical methods are appropriate | NA |
| 8. Provide a clear description of how missing items will be handled | The way missing items will be handled is clearly described | NA |
| ***Translation process*** | | |
| *Design requirements* | | |
| 1. Describe both the original language in which the PROM was developed, the source language (if different from the original language) and the language in which the PROM will be translated | Original language, source language and target language will be described | See Methods |
| 2. Ensure that the items will be translated forward and backward | Multiple forward and multiple backward translations | See Methods |
| 3. Ensure that both forward translators have a mother tongue in the target language in which the PROM will be translated | Both forward translators have a mother tongue in the target language in which the PROM will be translated | See Methods |
| 4. Ensure that one of the forward translators has expertise in the diseases involved, and in the construct measured by the PROM; the other forward translators is naïve on the construct measured by the PROM | One of the forward translators has expertise on disease and construct to be measured, another translator is naïve | See Methods |
| 5. Ensure that both backward translators have a mother tongue in the original or source language | Both forward translators have a mother tongue in the source language in which the PROM will be translated | See Methods |
| 6. Ensure that both backward translators are naïve in the disease involved and the construct to be measured | Both backward translators will be naïve in the disease involved and the construct to be measured | See Methods |
| 7. Ensure that the translators will work independently from each other | Translators will work independent | See Methods |
| 8. Provide a clear description on how differences between the original and translated versions will be resolved | Adequate description of how differences between translators will be resolved | See Methods |
| 9. Ensure that the translation will be reviewed by a committee (including the original developers of the PROM) | Translation will be reviewed by a committee (involving other people than the translators, e.g., the original developers) | See Methods |
| 10. Write a feedback report of the translation process | Feedback report will be written | See Methods |
| 11. Perform a pilot study (e.g., cognitive interview study) to check (1) the relevance of each item for the patients’ experience with the condition, AND   (2) the comprehensiveness of the PROM, AND  (3) the comprehensibility of the PROM instructions, items, response options, and recall period | Widely recognized or well justified method for qualitative research will be used to assess the three aspects | See Methods |
| 12. Perform the pilot study in a patient population representing the target population | The study will be performed in a sample representing the target population | See Methods |

* The context of use refers to the intended application of the PROM (e.g., for research or clinical practice), to a specific setting for which the PROM was developed (e.g., for use in a hospital or at home) or to a specific administration mode (e.g. paper or computer‐administered). If the PROM was developed for use across multiple contexts, this should be described.

**Legend:** NA, Not Applicable.
