# Supplementary material for: Measuring Reasons for Unfinished Nursing Care as Perceived by Community Care Nurses: A Binational Development and Validation Study
Source: Public Health Nurs. 2026 Jun 3;43(5):1394–408. doi: 10.1111/phn.70140 (PMC13535899; doi:10.1111/phn.70140)
Supplement: Supplementary file 7 [file PHN-43-1394-s007.docx]

**Supplementary Figure 1.** Scree plot on Israeli data

**Legend:** FA, Factor analysis; PC, Principal Component Analysis.

● and ◌: factors.
