# Supplementary material for: Measuring Reasons for Unfinished Nursing Care as Perceived by Community Care Nurses: A Binational Development and Validation Study
Source: Public Health Nurs. 2026 Jun 3;43(5):1394–408. doi: 10.1111/phn.70140 (PMC13535899; doi:10.1111/phn.70140)
Supplement: Supplementary file 8 [file PHN-43-1394-s008.docx]

**Supplementary Table 4.** Exploratory factor analysis in the entire data set comprising Israeli and Italian data

| **Items** | **Factor 1** | **Factor 2** | **Factor 3** | **Complexity** |
| --- | --- | --- | --- | --- |
| 1. Inadequate number of nurses | 0.2537 | 0.**6609** | -0.0302 | 1.2932 |
| 2. Inadequate number of administrative personnel | 0.2971 | **0.4499** | 0.0334 | 1.7461 |
| 3. Inadequate skill-mix (e.g., excessive number of nurses without advanced training compared to nurses with advanced training) | 0.1008 | -0.0258 | **0.7100** | 1.0430 |
| 4. Inadequate nursing care model (e.g., functional tasks-oriented model of care) | **0.4351** | **0.5337** | 0.1669 | 2.1401 |
| 5. Unbalanced workloads assigned across nurses during the shift | **0.3174** | **0.6518** | 0.1338 | 1.5466 |
| 6. Unbalanced nursing competences in the shift (e.g., excessive number of newly hired or non-expert nurses compared to experts) | -0.0472 | 0.1562 | **0.7741** | 1.0890 |
| 7. Unexpected rise in patient acuity/complexity | 0.2705 | **0.6001** | 0.0228 | 1.3936 |
| 8. Increased workload during the shift (e.g., increased numbers of patients at need) | 0.2461 | **0.7949** | -0.0478 | 1.1979 |
| 9. Inaccurate initial priority setting | **0.5108** | **0.5690** | 0.1691 | 2.1651 |
| 10. Inadequate priority re-assessment during the shift | **0.5068** | **0.5548** | 0.2451 | 2.3750 |
| 11. Inadequate skills/competences of nurses | 0.1473 | 0.0403 | **0.8766** | 1.0608 |
| 12. Inadequate confidence in performing skills by nurses | 0.1333 | 0.0023 | **0.8409** | 1.0502 |
| 13. Underuse or under recognition of the nursing competences | 0.1647 | 0.0491 | **0.7485** | 1.1056 |
| 14. Repeated interruptions of care activities (e.g., unplanned or unexpected activities) | **0.4114** | **0.5971** | 0.0362 | 1.7835 |
| 15. Medications prescribed not available | **0.6939** | **0.3094** | 0.1069 | 1.4369 |
| 16. Equipment not available/not functioning properly when needed | **0.6860** | **0.3656** | 0.1600 | 1.6531 |
| 17. Other multidisciplinary team did not provide the service expected (e.g., delays in obtaining the home advice for palliative care, mental health care) | **0.6959** | **0.4410** | 0.0534 | 1.7057 |
| 18. Caregiver unavailable (e.g., to inform him/her about the care to be continued at home) | **0.7218** | **0.3769** | -0.0289 | 1.5115 |
| 19. Incomplete or interrupted communication between the community nursing staff and the hospital nursing staff | **0.7388** | **0.3272** | 0.0679 | 1.3972 |
| 20. Incomplete or interrupted communication among the community nursing staff | **0.7952** | 0.2681 | 0.2191 | 1.3891 |
| 21. Incomplete or interrupted communication between nursing and medical staff | **0.8034** | 0.2623 | 0.0651 | 1.2252 |
| 22. Lack of support/collaboration among the community team members | **0.7423** | **0.3585** | 0.1432 | 1.5286 |
| 23. Excess time spent travelling to reach homes or the community centers | **0.4793** | 0.2510 | 0.2510 | 2.0844 |
| **Proportion Variance, %** | 25.67 | 18.97 | 15.05 |  |
| **Cumulative Variance, %** | 25.67 | 44.64 | 69.69 |  |
| **Cronbach's Alpha, factor level** | **0.9372** | **0.8997** | **0.8950** |  |
| **Total McDonald's Omega** | **0.9575** |  |  |  |
